# The clinical value of metabolic syndrome and its components with respect to sudden cardiac death using different definitions: Two decades of follow-up from the Tehran Lipid and Glucose Study

**DOI:** 10.1186/s12933-022-01707-1

**Published:** 2022-12-03

**Authors:** Soroush Masrouri, Seyyed Saeed Moazzeni, Neda Cheraghloo, Fereidoun Azizi, Farzad Hadaegh

**Affiliations:** 1 Prevention of Metabolic Disorders Research Center, Research Institute for Endocrine Sciences, Shahid Beheshti University of Medical Sciences, P.O. Box: 19395-4763, Tehran, 1985717413 Iran; 2 Endocrine Research Center, Research Institute for Endocrine Sciences, Shahid Beheshti University of Medical Sciences, Tehran, Iran; 3 Department of Epidemiology and Biostatistics, School of Public Health, Tehran University of Medical Sciences, Tehran, Iran

**Keywords:** Metabolic syndrome, Sudden cardiac death, Prospective cohort study, Tehran Lipid and Glucose Study

## Abstract

**Background:**

To evaluate the impact of different definitions of metabolic syndrome (MetS) and their components on the risk of sudden cardiac death (SCD) among the Iranian population according to the World Health Organization (WHO), International Diabetes Federation (IDF), Adult Treatment Panel III (ATP III), and Joint Interim Statement (JIS) criteria.

**Methods:**

The study population included a total of 5,079 participants (2,785 women) aged ≥ 40 years, free of cardiovascular disease (CVD) at baseline. Participants were followed for incident SCD annually up to 20 March 2018**.** Multivariable Cox proportional hazards regression models were applied to estimate the hazard ratios (HRs) and 95% confidence intervals (CIs) of MetS and its components for incident SCD.

**Results:**

The prevalence of MetS ranged from 27.16% to 50.81%, depending on the criteria used. Over a median of 17.9 years of follow-up, 182 SCD events occurred. The WHO, IDF, and JIS definitions were strong predictors of SCD with multivariable-adjusted HRs (95% CI) of 1.68 (1.20–2.35), 1.51 (1.12–2.03), and 1.47 (1.08–1.98), respectively; these associations significantly attenuated after further adjustment for MetS components. MetS by the ATP III definition was not associated with the risk of SCD after controlling for antihypertensive, glucose-lowering, and lipid-lowering medication use. Among the components of MetS, high blood pressure (WHO definition), high waist circumference (using the national cutoff of ≥ 95 cm), and high glucose component by the JIS/IDF definitions remained independent predictors of SCD with HRs of 1.79 (1.29–2.48), 1.46 (1.07–2.00), and 1.52 (1.12–2.05), respectively.

**Conclusions:**

The constellation of MetS, except for when defined with ATP III definition, is a marker for identifying individuals at higher risk for SCD; however, not independent of its components. Among MetS components, abdominal obesity using the population-specific cutoff point, high glucose component (JIS/IDF definitions), and high blood pressure (WHO definition) were independent predictors of SCD.

**Supplementary Information:**

The online version contains supplementary material available at 10.1186/s12933-022-01707-1.

## Introduction

The concept of metabolic syndrome (MetS) refers to the clustering of metabolic abnormalities, including dysglycemia, obesity, elevated blood pressure, and dyslipidemia [1, 2]. MetS has developed into a worldwide epidemic in recent decades with markedly increasing trends [3]. Based on the National Health and Nutrition Examination Survey (NHANES, 2011 to 2016) data, the prevalence of MetS among US adults is estimated at 34.7% [4]. MetS affects about one-third of the Iranian adults [5], and roughly 5% of the Tehranian adults develop the syndrome each year [6]. Although there is considerable debate regarding what constitutes MetS, its association with poor outcomes such as type 2 diabetes (T2D), cardiovascular disease (CVD), and coronary heart disease (CHD) is well-accepted in the clinical setting [7–10]. Individuals with MetS exhibit more than a twofold higher risk of CVD and cardiovascular mortality and a 1.5-fold higher risk of total mortality [7].

More than 50% of all CVD deaths are attributable to sudden cardiac death (SCD), which accounts for up to 230,000–350,000 deaths in the US annually [11]. SCD refers to the sudden and unexpected death from a cardiac cause that generally happens within ≤ 1 h of symptom onset when witnessed or, if unwitnessed, within 24 h of the subject last seen to be alive and well before the event [12, 13]. Often, SCD is the first recognized clinical sign of underlying heart disease, which occurs unanticipated [14]. Overall survival after sudden cardiac arrest is poor in the afflicted individuals due to the need for immediate cardiopulmonary resuscitation (CPR), defibrillation after the event, and post-arrest care, which require enormous medical resources and trained individuals [15, 16]. These challenges highlight the importance of identifying traditional risk factors and adopting screening programs for people at a high-risk state for SCD who can benefit from primary prevention. Understanding and managing SCD requires more attention in the Middle East and North Africa (MENA) region due to the high burden of CVD and its traditional risk factors [17–20]. Earlier, we found that more than 0.2% of the Iranian population experience SCD each year [21].

There is a well-established link between classical cardiovascular risk factors, including high blood pressure (BP), elevated fasting plasma glucose (FPG), obesity, and dyslipidemia with cardiac alterations that can lead to SCD [22, 23]. Moreover, growing evidence demonstrate a possible association between MetS and SCD; a few population-based studies previously have applied different definitions for MetS and reported that MetS is accompanied by about a 50–160% increased risk of SCD, depending on the definition used [24–27]. However, it remains unclear whether the relationship between MetS and SCD is attributable to the MetS concept or its impact is driven solely by its components. Additionally, limited data exist regarding the agreement between different definitions of MetS with SCD, which are inconclusive since they were not usually adopted in the same population. Additionally, two of the previous studies only enrolled middle-aged men [24, 26], and these investigations were conducted among US, European, and East Asian populations [24–27].

Being the first research in the MENA region, over about two decades of follow-up in the large and prospective Tehran Lipid and Glucose Study (TLGS), we aimed to determine (1) the association of MetS and its components as defined by four different criteria with incident SCD, and (2) whether MetS per se has any excess risk independent of its components.

## Materials and methods

### Study design and population

The Tehran Lipid and Glucose Study (TLGS) is a prospective community-based cohort study performed on a representative sample of the Tehran urban population aged 3 years and older. This cohort study was initially established to determine the noncommunicable diseases (NCDs) prevalence and incidence. It also looked at related risk factors for NCDs and aimed to promote developing a healthy lifestyle to act against these risk factors. Recruiting participants for TLGS was conducted in two phases [the first (January 1999–August 2001: n = 15,005) and the second (October 2001–September 2005: n = 3,550)]. Data collection from this population is ongoing and planned to continue for a minimum of 20 years with 3-year intervals (i.e., 3rd phase: 2005 to 2008, 4th phase: 2009 to 2011, 5th phase: 2012 to 2015, and 6th phase: 2015 to 2018). The details of registration and design of the TLGS have been described before [28].

For the present study, as shown in Additional file 1: Figure S1, a total of 6,295 individuals aged ≥ 40 years (5282 from the first phase and 1013 from the second phase) were selected. Firstly, 569 participants with prevalent CVD at baseline were excluded, leading to 5726 participants. We also excluded those with missing data on MetS components or confounders (n = 196, considering overlap features). Finally, after further exclusion of 451 individuals without any follow-up data, 5079 participants remained eligible for the current analysis. For the analysis by the World Health Organization (WHO) criteria for MetS, further 139 individuals were excluded due to missing data on 2-h post-challenge plasma glucose (2 h-PG), leading to 4940 eligible participants.

### Clinical and laboratory measurements

TLGS questionnaires were used during the enrollment phases to collect demographics, past medical history of CVD, family history of premature CVD (FH-CVD), medication use, and smoking habits. Body weight of participants was measured using digital weight scales (Seca 707, Seca Corp., Hanover, MD, USA; range 0.1–150 kg) with light clothing on and shoes removed. Height was measured using a tape meter while individuals were in the normal standing position and did not have shoes on. Waist circumference (WC) was measured with a tape meter placed at the umbilical level without putting pressure on the body surface. Hip circumference (HC) at the maximal level of the hip over light clothing was measured. Body mass index (BMI) and waist to hip ratio (WHR) were calculated as weight divided by the square of height (kg/m^2^) and WC divided by HC, respectively.

According to the TLGS design [28], after a 15-min resting time in a sitting position, individuals' BP was recorded using a standard mercury sphygmomanometer as the mean of measurements taken two times on the right arm. Resting heart rate (RHR) was the mean of two times measuring the radial artery pulse in 1 min and was not based on electrocardiogram analysis. A blood sample was collected to measure biochemical parameters from all participants after an overnight fasting period of at least 12 h before morning blood collection. Taken samples were then analyzed at the TLGS research laboratory on the blood collection day. For the 2 h-PG, subjects without known diabetes took 82.5-g glucose monohydrate solution (an equivalent of 75 g anhydrous glucose), and a blood sample was taken 2 h afterward. Details of lipid measurements, including triglycerides (TG) and high-density lipoprotein cholesterol (HDL-C), are described elsewhere [28].

### Definition of terms

FH-CVD was defined as a prior diagnosis of CVD in male (aged < 55 years) or female (aged < 65 years) first-degree blood relatives. Smoking was defined as any record of smoking at the time of examination. Self-reported CVD was defined as a “yes” answer to the question of "whether the individual has ever had a prior diagnosis of CVD by a physician, i.e., either prior ischemic heart disease (IHD), confirmed myocardial infarction (MI), CCU admission, history of cerebrovascular accident (CVA), intermittent claudication, angiography, or angioplasty”. T2D was defined as treatment with glucose-lowering agents or having FPG ≥ 126 mg/dl or 2 h-PG ≥ 200 mg/dl at the enrollment phase.

### Definition of the metabolic syndrome

Criteria used for defining MetS and cutoff points for their components, including the WHO [29], IDF (International Diabetes Federation) [30], ATP III (Adult Treatment Panel III) [31], and JIS (Joint Interim Statement) [32], are summarized in Table 1. The report of the Iranian National Committee of Obesity recommended that WC criteria follow the appropriate country- and population-specific cutoff points; thus, abdominal obesity for the IDF and JIS definitions was defined as having a WC of ≥ 95 cm for both men and women [33, 34]. However, in the present analysis, we used ATP III criteria with the original WC cutoffs (> 102 cm for men and > 88 cm for women) [31]. As recommended by the European Group for the Study of Insulin Resistance (EGIR) [35], microalbuminuria from the WHO definition was excluded for the usage in the epidemiological studies [36]. Therefore, a modified version of the WHO criteria was used in the present study than the proposed version [29]; accordingly, measuring urine albumin excretion and serum insulin was disregarded.

**Table 1:** Metabolic syndrome defined by the modified WHO, IDF, ATP III, and JIS diagnostic criteria

The modified WHO definition [29]	The IDF definition [30]	The ATP III definition [31]	The JIS definition [32]
DM or IGT [2 h-PG ≥ 140 mg/dl (7.8 mmol/l)] Plus two or more of the following:	WC ≥ 95 cm Plus any two or more of the following:	Three or more of the following:	Three or more of the following:
–	FPG ≥ 100 mg/dl (5.6 mmol/l) or drug treatment	FPG ≥ 110 mg/dl (6.1 mmol/l)	FPG ≥ 100 mg/dl (5.6 mmol/l) or drug treatment
TG ≥ 150 mg/dl (1.7 mmol/l) or M: HDL-C < 35 mg/dl (0.9 mmol/l) F: HDL-C < 39 mg/dl (1.0 mmol/l)	TG ≥ 150 mg/dl (1.7 mmol/l) or drug treatment	TG ≥ 150 mg/dl (1.7 mmol/l)	TG ≥ 150 mg/dl (1.7 mmol/l) or drug treatment
	M: HDL-C < 40 mg/dl (1.03 mmol/l) F: HDL-C < 50 mg/dl (1.29 mmol/l) or drug treatment	M: HDL-C < 40 mg/dl (1.03 mmol/l) F: HDL-C < 50 mg/dl (1.29 mmol/l)	M: HDL-C < 40 mg/dl (1.03 mmol/l) F: HDL-C < 50 mg/dl (1.29 mmol/l) or drug treatment
BMI > 30 kg/m^2^ or M: WHR > 0.90 F: WHR > 0.85	-	M: WC > 102 cm F: WC > 88 cm	WC ≥ 95 cm
BP ≥ 140/90 mmHg or drug treatment	BP ≥ 130/85 mmHg or drug treatment	BP ≥ 130/85 mmHg	BP ≥ 130/85 mmHg or drug treatment

*JIS* Joint Interim Statement, *IDF* International Diabetes Federation, *ATP III* Adult Treatment Panel III, *WHO* World Health Organization, *FPG* fasting plasma glucose, *DM* diabetes mellitus, *IGT* impaired glucose tolerance, *2 h-PG* 2-h post-challenge glucose, *WHR* waist to hip ratio, *WC* waist circumference, *BMI* body mass index, *TG* triglycerides, *HDL-C* high-density lipoprotein cholesterol, *BP* blood pressure, *M* male, *F* female

### Adjudication of sudden cardiac death

Outcome assessments of the TLGS have been published elsewhere in detail [28, 37]. All individuals were followed up for any medical events annually via a phone call. A trained nurse called individuals and recorded medical events leading to hospitalization. Then, during a house or hospital visit, individuals were followed up on any reported event and were asked for complementary medical documents by a trained physician. Moreover, information regarding death certificates, forensic reports, and, where possible, verbal autopsies were gathered for those who died. Verbal autopsies were carried out by trained nurses as a secondary interview with other surviving family members using predefined questions to gather further information regarding medical history, signs and symptoms preceding death in order to help distinguish different causes of death [38]. Collected documents were then investigated by an outcome committee comprised of a principal investigator, an internist, an endocrinologist, a cardiologist, an epidemiologist, and other experts in the case of necessity. Fatal cases in the TLGS were crucially assessed by the outcome committee members; after adjudication by the outcome committee, each event was attributed to a specific outcome. Definite SCD was verified as a sudden pulseless condition attributable to a cardiac origin in a previously stable individual. Possible SCD was defined as unpredictable death, 24 h after last witnessed to be alive and well, that was not attributable to a specific source of circulatory collapse or underlying sources other than cardiac diseases. For this study, both cases of definite and possible SCD were included in the final analysis [39, 40].

### Statistical analysis

Participants’ characteristics were presented as mean ± SD and frequencies (%) for continuous and categorical variables, respectively. In the case of a highly skewed distribution (e.g., TG), descriptive statistics were summarized as median (interquartile range: IQR)**.** Baseline characteristics were compared between responders (study participants) and non-responders (those with missing data on MetS components, covariates, and those without any follow-up data). Moreover, baseline characteristics of the study participants were described among subjects with and without MetS at baseline, and also among those with and without outcome (SCD) occurrence during follow-up. The Student *t*-test, Kruskal Wallis test, and χ2 test were used as appropriate.

Kaplan–Meier analyses were performed to compare the risk of SCD between subjects with and without MetS by different definitions of MetS. The multivariable Cox proportional hazards regression model was used to estimate hazard ratios (HRs) with 95% confidence intervals (CIs) of the SCD events according to the baseline MetS status and each component of MetS for different definitions. For all analyses, model 1 was adjusted for sex and age; model 2 was further adjusted for smoking, RHR, and FH-CVD; for the ATP III and WHO definitions that did not include medication use, model 3 was further adjusted for using medications [lipid-lowering, and glucose-lowering medications for both definitions and antihypertensive medication use only for ATP III definition]; model 4 was additionally adjusted for MetS components. The proportional hazard assumptions in the Cox models were checked using Schoenfeld's global test of residuals, and all proportionality assumptions were generally appropriate. Time to event is defined as the time of censoring or the SCD occurring, whichever came first. We censored subjects in the case of leaving the district, if they were lost to follow-up, died of a cause other than SCD, or were alive in the study until the end of the study (March 20, 2018; Additional file 1: Figure S1). Statistical analyses were preformed using the STATA version 14 (StataCorp LP, College Station, Texas) statistical software. A 2-tailed P-value of less than 0.05 was considered statistically significant for all analyses.

## Results

Baseline characteristics of responders and non-responders are reported in Additional file 3: Table S1. Responders were generally younger and were less on glucose- or lipid-lowering medications than the non-responders; however, non-responders had a higher level of HDL-C.

The study population included a total of 5079 participants (women = 2785) with a mean age (SD) of 53.63 (9.94). Among the total population, the prevalence of MetS at baseline was 50.81%, 35.08%, 45.21%, and 27.16%, based on the JIS, IDF, ATP III, and WHO criteria, respectively.

As shown in Table 2, all baseline characteristics of the study population differed significantly by the presence of JIS-MetS; as predicted, in subjects with MetS, the cardiometabolic profile was worse than in the non-MetS group, excluding smoking, which was less prevalent among those with MetS.

**Table 2:** Baseline characteristics of the study population stratified by the presence of metabolic syndrome (JIS): Tehran Lipid and Glucose Study (1999–2018)

	Overall	Non-MetS	MetS	P-value
Number of participants	5079	2498	2581	
Continuous variables, Mean ± SD				
Age (year)	53.63 ± 9.94	52.31 ± 10.12	54.92 ± 9.60	< 0.01
BMI (kg/m^2^)	27.88 ± 4.60	26.01 ± 4.10	29.68 ± 4.33	< 0.01
WC (cm)	92.71 ± 11.22	87.08 ± 9.60	98.16 ± 9.91	< 0.01
WHR	0.91 ± 0.08	0.88 ± 0.08	0.94 ± 0.08	< 0.01
SBP (mmHg)	126.11 ± 20.90	117.70 ± 17.56	134.26 ± 20.64	< 0.01
DBP (mmHg)	80.10 ± 11.43	75.48 ± 10.02	84.56 ± 10.93	< 0.01
RHR (beat/min)	78.34 ± 11.44	77.20 ± 11.14	79.45 ± 11.61	< 0.01
FPG (mg/dl)	105.18 ± 39.38	92.95 ± 22.98	117.02 ± 47.50	< 0.01
HDL-C (mg/dl)	41.67 ± 10.95	44.64 ± 11.64	38.80 ± 9.38	< 0.01
TG (mg/dl)	165 (115–233)*	122 (91–161)*	210 (165–280)*	< 0.01
Categorical variables, number (%)				
Men	2294 (45.17)	1249 (50)	1045 (40.49)	< 0.01
Current smoking, yes	803 (15.81)	481 (19.26)	322 (12.48)	< 0.01
Family History of premature CVD, yes	932 (18.35)	418 (16.73)	514 (19.91)	< 0.01
Glucose-lowering drug use, yes	335 (6.60)	55 (2.20)	280 (10.85)	< 0.01
Antihypertensive drug use, yes	569 (11.20)	98 (3.92)	471 (18.25)	< 0.01
Lipid-lowering drug use, yes	252 (4.96)	24 (0.96)	228 (8.83)	< 0.01

*SD* standard deviation, *BMI* body mass index, *WC* waist circumference, *WHR* waist to hip ratio, *SBP* systolic blood pressure, *DBP* diastolic blood pressure, *RHR* resting heart rate, *FPG* fasting plasma glucose, *HDL-C* high-density lipoprotein cholesterol, *CVD* cardiovascular disease

^*^Data presented as median (IQR)

Baseline characteristics of the participants (for the JIS/IDF/ATP III criteria) according to the occurrence of SCD during the follow-up period are shown in Table 3. Compared to those who did not have an SCD event till the end of the study, those with incident SCD were mainly men, older, more likely to smoke, and had higher levels of FPG, systolic (SBP) and diastolic blood pressure (DBP), WC, and WHR, while RHR, FH-CVD prevalence, TG, HDL-C, and BMI levels did not differ between the two groups. Baseline characteristics of the participants for the WHO criteria are shown in Additional file 4: Table S2.

**Table 3:** Baseline characteristics of the study population stratified by the incidence of sudden cardiac death (total population): Tehran Lipid and Glucose Study (1999–2018)

	**Without SCD**	**With SCD**	**P-value**
Number of participants	4897	182	
Continuous variables, Mean ± SD			
Age (year)	53.30 ± 9.77	62.62 ± 10.16	< 0.01
BMI (kg/m^2^)	27.89 ± 4.58	27.48 ± 5.04	0.24
WC (cm)	92.63 ± 11.20	94.94 ± 11.87	< 0.01
WHR	0.91 ± 0.08	0.96 ± 0.08	< 0.01
SBP (mmHg)	125.70 ± 20.59	137.45 ± 25.58	< 0.01
DBP (mmHg)	80.00 ± 11.30	82.70 ± 14.29	< 0.01
RHR (beat/min)	78.35 ± 11.40	78.18 ± 12.80	0.85
FPG (mg/dl)	104.36 ± 37.91	127.28 ± 64.10	< 0.01
HDL-C (mg/dl)	41.69 ± 10.94	41.18 ± 11.26	0.54
TG (mg/dl)	165 (115–233)*	166 (118–235)*	0.90
Categorical variables, number (%)			
Men	2174 (44.39)	120 (65.93)	< 0.01
Current smoking, yes	761 (15.54)	42 (23.08)	< 0.01
Family History of premature CVD, yes	905 (18.48)	27 (14.84)	0.21
Glucose-lowering drug use, yes	302 (6.17)	33 (18.13)	< 0.01
Antihypertensive drug use, yes	527 (10.76)	42 (23.08)	< 0.01
Lipid-lowering drug use, yes	239 (4.88)	13 (7.14)	0.17

*SD* standard deviation, *BMI* body mass index, *WC* waist circumference, *WHR* waist to hip ratio, *SBP* systolic blood pressure, *DBP* diastolic blood pressure, *RHR* resting heart rate, *FPG* fasting plasma glucose, *HDL-C* high-density lipoprotein cholesterol, *CVD* cardiovascular disease

^*^Data presented as median (IQR)

**Fig. 1:**
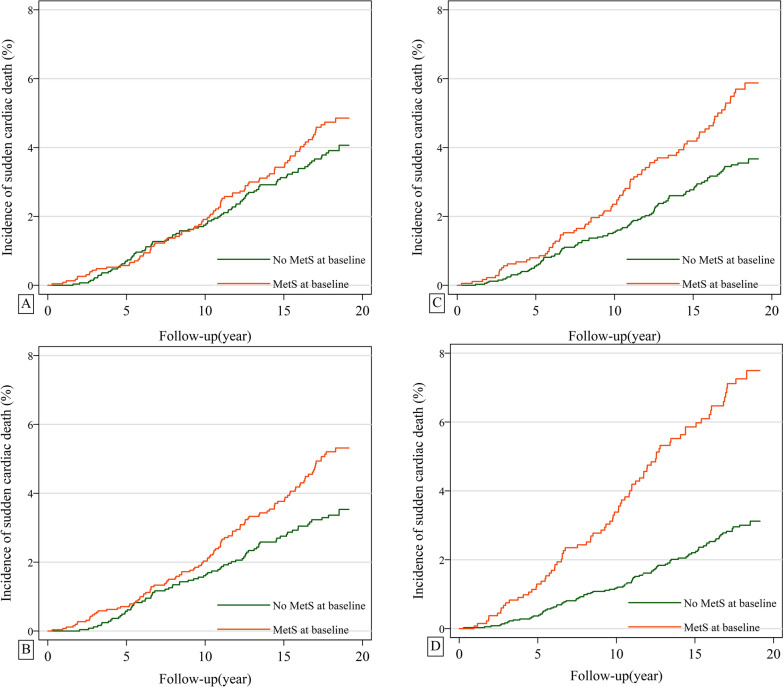
Cumulative incidence of sudden cardiac death by metabolic syndrome status at baseline according to the ATP III **A**, JIS **B**, IDF **C**, and WHO **D** criteria

During a median 17.9-year follow-up (interquartile range: 13.6–18.5 years), we documented 182 and 171 cases of incident SCD for the JIS-/IDF-/ATP III-MetS and the WHO-MetS, respectively. Deaths due to other causes among the study population are shown in Additional file 2: Figure S2. The cumulative incidence of SCD for different definitions of MetS is shown in Fig. 1. Generally, those with MetS at baseline had higher rate of incident SCD, the difference was most prominent for the WHO-MetS and least significant for ATP III-MetS.

Table 4 shows adjusted HRs and 95% CIs of the MetS defined by the four criteria for the SCD incidence. MetS increased the risk of SCD with age- and sex-adjusted HRs of 2.07 (95% CI 1.53–2.80), 1.50 (1.12–2.02), 1.42 (1.05–1.92), and 1.34 (0.99–1.81, P-value: 0.06), for the definitions of WHO, IDF, JIS, and ATP III, respectively. After further adjustment for smoking, RHR, and FH-CVD in model 2, these associations remained significant, with HRs of 2.10 (1.55–2.86) for the WHO, 1.51 (1.12–2.03) for the IDF, 1.47 (1.08–1.98) for the JIS, and 1.36 (1.00–1.85) for the ATP III definition. Regarding the ATP III and WHO MetS definitions, in model 3, after further adjustments with medication use, only WHO-MetS remained a significant predictor of SCD [HR, 95% CI 1.68 (1.20–2.35)]. However, after additional adjustment for the components of MetS, all of the above associations attenuated to the non-significant p-values.

**Table 4:** HRs and 95% CIs showing the relationship of different definitions of metabolic syndrome with incidence of sudden cardiac death: Tehran Lipid and Glucose Study (1999–2018)

	E/N	Model 1		Model 2		Model 3		Model 4	
		HR (95% CI)	P-value	HR (95% CI)	P-value	HR (95% CI)	P-value	HR (95% CI)	P-value
MetS (JIS)	109/2581	1.42 (1.05–1.92)	0.02	1.47 (1.08–1.98)	0.01	–	–	0.88 (0.50–1.55)	0.66
MetS (IDF)	82/1782	1.50 (1.12–2.02)	0.01	1.51 (1.12–2.03)	0.01	–	–	0.74 (0.40–1.38)	0.34
MetS (ATP III)	89/2296	1.34 (0.99–1.81)	0.06	1.36 (1.00–1.85)	0.05	1.06 (0.76–1.47)	0.73	0.81 (0.46–1.42)	0.36
MetS (WHO)	80/1342	2.07 (1.53–2.80)	< 0.01	2.10 (1.55–2.86)	< 0.01	1.68 (1.20–2.35)	< 0.01	1.23 (0.58–2.62)	0.59

Model 1: Adjusted for age + sex

Model 2: Model 1 + smoking + resting heart rate + family history of premature CVD

Model 3: Model 2 + antihypertensive medications (only for ATP III definition) + lipid-lowering medications + glucose-lowering medications

Model 4: Further adjustment with MetS components

*HR* hazard ratio, *CI* confidence interval, *MetS* metabolic syndrome, *JIS* joint interim statement, *IDF* International Diabetes Federation, *ATP III* Adult Treatment Panel III, *WHO* World Health Organization

*N* Subjects with metabolic syndrome for each definition

*E* Number of SCD events

Table 5 shows the adjusted HRs and 95% CIs of SCD for individual MetS components according to different definitions. Of the JIS-/IDF-MetS components, FPG ≥ 100 mg/dl and WC ≥ 95 cm increased the risk of SCD by more than 60% in models 1 and 2; after further adjustment for other MetS components, HRs for FPG ≥ 100 mg/dl and WC ≥ 95 cm were 1.52 (95% CI 1.12–2.05) and 1.46 (1.07–2.00), respectively. After applying the cutoff points of ATP III-MetS, FPG ≥ 110 mg/dl in models 1 and 2 and BP ≥ 130/85 mmHg only in model 2 were significant predictors of SCD; after further adjustments with medications (model 3), none of these associations remained statistically significant. Regarding the WHO-MetS, HRs for BP ≥ 140/90 mmHg (or drug treatment), dysglycemia, obesity, and dyslipidemia were 1.96 (1.43–2.69), 1.95 (1.44–2.65), 1.65 (1.07–2.54), and 1.43 (1.03–2.00), after adjustment for confounders in model 2, respectively; only the first two components remained significant predictors of SCD after adjustment for medication use in model 3 [HR, 95% CI 1.92 (1.40–2.64) and 1.49 (1.06–2.10), respectively]. In the fully adjusted model (model 4), the association of BP ≥ 140/90 mmHg remained significant [HR, 95% CI 1.79 (1.29–2.48)].

**Table 5:** HRs and 95% CIs showing the relationship between metabolic syndrome components and incidence of sudden cardiac death: Tehran Lipid and Glucose Study (1999–2018)

	E/N	Model 1		Model 2		Model 3		Model 4	
		HR (95% CI)	P-value	HR (95% CI)	P-value	HR (95% CI)	P-value	HR (95% CI)	P-value
The JIS/IDF									
High WC	100/2219	1.62 (1.20–2.17)	< 0.01	1.62 (1.21–2.18)	< 0.01	–	–	1.46 (1.07–2.00)	0.02
High FPG	88/1742	1.63 (1.22–2.18)	< 0.01	1.66 (1.24–2.23)	< 0.01	–	–	1.52 (1.12–2.05)	0.01
High BP	114/2439	1.24 (0.91–1.70)	0.17	1.34 (0.98–1.84)	0.07	–	–	1.17 (0.84–1.61)	0.35
High TG	111/2974	1.25 (0.93–1.70)	0.15	1.26 (0.93–1.71)	0.13	–	–	1.12 (0.81–1.56)	0.50
Low HDL-C	114/3594	0.90 (0.67–1.22)	0.51	0.88 (0.65–1.20)	0.40	–	–	0.78 (0.57–1.08)	0.13
The ATP III									
High WC	70/2207	1.31 (0.92–1.86)	0.07	1.30 (0.91–1.86)	0.15	1.12 (0.78–1.61)	0.55	1.04 (0.72–1.53)	0.81
High FPG	61/998	2.02 (1.49–2.76)	< 0.01	2.06 (1.51–2.81)	< 0.01	1.35 (0.92–1.99)	0.13	1.30 (0.87–1.94)	0.20
High BP	112/2333	1.30 (0.96–1.77)	0.09	1.39 (1.02–1.91)	0.04	1.23 (0.89–1.69)	0.22	1.18 (0.85–1.63)	0.32
High TG	109/2938	1.24 (0.92–1.67)	0.16	1.25 (0.92–1.68)	0.15	1.12 (0.83–1.52)	0.46	1.14 (0.82–1.58)	0.43
Low HDL-C	113/3536	0.93 (0.69–1.26)	0.66	0.90 (0.67–1.22)	0.52	0.81 (0.60–1.10)	0.18	0.77 (0.56–1.06)	0.11
The WHO									
Dysglycemia	90/1691	1.91 (1.41–2.58)	< 0.01	1.95 (1.44–2.65)	< 0.01	1.49 (1.06–2.10)	0.02	1.31 (0.92–1.86)	0.14
Obesity	147/3748	1.65 (1.06–2.54)	0.02	1.65 (1.07–2.54)	0.02	1.49 (0.96–2.30)	0.07	1.22 (0.77–1.91)	0.40
High BP	93/1636	1.82 (1.33–2.49)	< 0.01	1.96 (1.43–2.69)	< 0.01	1.92 (1.40–2.64)	< 0.01	1.79 (1.29–2.48)	< 0.01
Dyslipidemia	121/3223	1.45 (1.04–2.02)	0.03	1.43 (1.03–2.00)	0.03	1.34 (0.96–1.87)	0.08	1.15 (0.82–1.63)	0.42

Model 1: Adjusted for age + sex

Model 2: Model 1 + smoking + resting heart rate + family history of premature CVD

Model 3: Model 2 + antihypertensive medications (only for ATP III definition) + lipid-lowering medications + glucose-lowering medications

Model 4: Further adjustment with MetS components

*HR* hazard ratio, *CI* confidence interval, *MetS* metabolic syndrome, *JIS* joint interim statement, *IDF* International Diabetes Federation, *ATP III* Adult Treatment Panel III, *WHO* World Health Organization, *WC* waist circumference, *FPG* fasting plasma glucose, *BP* blood pressure, *TG* triglycerides, *HDL-C* high-density lipoprotein cholesterol

N: Subjects with each component for each definition of metabolic syndrome

E: Number of SCD events

## Discussion

To our knowledge, this is the first community-based prospective study investigating the association between MetS and its components with incident SCD in the MENA region with a high burden of MetS. In the multivariable analysis, during about two decades of follow-up, we found that subjects with MetS by the JIS, IDF, and WHO were more likely to develop SCD than individuals without the syndrome at baseline; however, after controlling for MetS components, the concept of MetS did not remain a risk factor for SCD. Regarding MetS components, abdominal obesity (using the Iranian national cut-off point), elevated FPG for the JIS/IDF criteria, and elevated BP for the WHO criteria were independently associated with the risk of SCD.

Regarding SCD, the observed 1.4- to 1.6-fold increased risk associated with the MetS by either JIS, IDF, and WHO criteria is generally in line with other population-based studies conducted among Finnish [24], US [25], and French populations [26]. However, regarding the ATP III-MetS (2001) in our study, despite the increased risk shown in other studies [24, 26], we did not find any risk of SCD for this definition, after controlling for antihypertensive, glucose-lowering, and lipid-lowering medication use. The association of MetS with incident SCD among conducted studies is summarized in Table 6. It should be noted that comparison of the conducted studies is difficult due to the differences in methodology, criteria for defining MetS, levels of adjustment for confounders, and population characteristics. In a study including middle-aged Eastern Finnish men, all the abovementioned definitions were associated with a 2.2- to 2.6-fold excess risk of SCD [24]. Also, in accordance with our findings, Hess et al. [25] found that JIS-MetS significantly predicted SCD in 13,168 participants from the Atherosclerosis Risk in Communities (ARIC) study. Likewise, Empana et al. [26] found that among middle-aged French men, ATP III- and IDF-MetS were associated with excess risk of SCD after adjustment for confounders; however, in their study, HDL-C and WC were not included in the MetS definitions and no adjustments for the use of glucose-lowering and lipid-lowering medications were conducted. Moreover, based on the Korean National Health Insurance Service (K-NHIS) database, Kim et al. [27] found that among Korean participants (aged ≥ 20 years), MetS by the updated ATP III criteria, which applies a lower threshold for elevated FPG (≥ 100 mg/dl) and includes medication use, was associated with a 50.7% increased SCD risk. In the current data set, we found that subjects with MetS did not have an increased risk for SCD, independent of their components. In line with our findings, Hess et al. [25] highlighted that JIS-MetS did not alter the risk of SCD after controlling for MetS components.

**Table 6:** Association of metabolic syndrome with the incidence of sudden cardiac death in retrospective/prospective

Study/ Authors Name	Follow-up, y	Age, y	Country	Sample size, Number	SCD, Number	Adjustments	MetS criteria	RR/HR (95% CI)
Paris Prospective Study I: 2007 [26]	21.2	Middle -age	France	M: 6678	105	Age, mean number of cigarettes smoked daily in the past 5 years, sporting activity, total cholesterol, family history of sudden death, and fatal myocardial infarction	ATP III (2001)	HR: 1.68 (1.05–2.70)
							IDF	HR: 2.02 (1.30–3.14)
KIHD Study: 2016 [24]	21.5	Middle -age	Finland	M: 1466	85	Age and examination year, socio-economic status, smoking, alcohol consumption, family history of coronary heart disease, LDL cholesterol concentrations, dietary intake of saturated fats, and energy expenditure of leisure-time physical activity	WHO	RR: 2.34 (1.45–3.78)
							ATP III (2001)	RR: 2.50 (1.36–4.58)
							IDF	RR: 2.20 (1.20–4.04)
							JIS	RR: 2.57 (1.63–4.06)
ARIC Study: 2017 [25]	23.6	54 (49–59)*	US (24.8% blacks)	F: 7374 M: 5794	357	Age, sex, race, heart rate, smoking status, use of calcium channel blockers, b-blockers, and antiarrhythmic medications, left ventricular hypertrophy	JIS	HR: 1.70 (1.37–2.12)
Kim Study: 2022 [27]	2009 -2018	≥ 20	Korea	F: 1,822,892 M: 2,233,531	16,352	Age, sex, body mass index, alcohol consumption, smoking status, regular physical activity, and income level	ATP III (2005)	HR: 1.51 (1.46–1.56)
TLGS (Present study)	17.9	52 (45–61)*	Iran	F: 2785 M: 2294	WHO-MetS:171 JIS-/IDF-/ATP III-MetS:182	Age, sex, current smoking, resting heart rate, family history of premature cardiovascular disease ^†^	WHO	HR: 1.68 (1.20–2.35)
							ATP III (2001)	HR: 1.06 (0.76–1.47)
							IDF	HR: 1.51 (1.12–2.03)
							JIS	HR: 1.47 (1.08–1.98)

*SCD* sudden cardiac death, *MetS* metabolic syndrome, *RR* relative risk, *HR* hazard ratio, *JIS* Joint Interim Statement, *IDF* International Diabetes Federation, *ATP III* Adult Treatment Panel III, *WHO* World Health Organization, *KIHD* Kuopio Ischemic Heart Disease, *ARIC* Atherosclerosis Risk in Communities, *TLGS* Tehran Lipid and Glucose Study

^*^Median (interquartile range)

^†^The ATP III and WHO definitions were further adjusted for using medications [lipid-lowering, and glucose-lowering medications for both definitions and antihypertensive medication use only for ATP III definition]

When we considered individual components of MetS, obesity, as defined by BMI > 30 kg/m^2^ or elevated WHR, by the WHO definition, was associated with a 65% increased risk of SCD in the multivariable analysis; however, this association did not remain significant after controlling for medication use and other MetS components. We also found that subjects with abdominal obesity (WC ≥ 95 cm) had 1.46 times greater risk of incident SCD, independent of other MetS components. Considering ATP III definition, WC > 102 cm for men and > 88 cm for women, contrary to the ARIC study [25], was not associated with the risk of SCD. These findings further support Alberti and colleagues’ [32–34] recommendation that the appropriate cutoff point of WC in MetS definition should be population-specific and defined according to hard outcomes in each population.

In this study, after controlling for other MetS components, 52% higher risk of incident SCD was observed for high FPG criteria (JIS/IDF definitions). However, for FPG ≥ 110 mg/dl according to the ATP III definition, after controlling for antihypertensive, glucose-lowering, and lipid-lowering medication use, no association with the SCD risk was found. Previously, in a systematic review and meta-analysis, it was found that for CVD and total mortality, both of these cutoff points for prediabetes were associated with higher risk; however, the effect of FPG ≥ 110 mg/dl was more prominent [41]. Moreover, Aune et al. [42], in a systematic review and meta-analysis of 19 population-based prospective studies, found that prediabetes and diabetes are associated with a 23% and a twofold increased risk of SCD (I^2^ = 0%, 6%), respectively.

In our data set, the high BP component according to the WHO-MetS, but not BP ≥ 130/85 mmHg according to the JIS/IDF/ATP III-MetS, independent of other cardiometabolic risk factors, increased the risk of SCD by 79%. Considering existing evidence from the Paris Prospective Study I [26], BP ≥ 130/85 mmHg (or drug treatment) was not significantly associated with the risk of SCD. In contrast, in the ARIC study, Hess et al. [25] reported an approximately 80% excess risk of SCD for the same threshold. Rapsomaniki et al. [43], in a cohort study of 1.25 million people free of CVD at baseline, showed that among those aged 30–59 years, compared to SBP of 115 mmHg, increased risk of cardiac arrest/SCD was observed for SBP cutoffs of 140–159 mmHg, but not for SBP cutoffs of 130–139 mmHg. Similarly, Dorjgochoo et al. [44], in a prospective study of 68,438 Chinese women aged 40–70 years, found that in contrast to BP ≥ 140/90 mmHg, high normal BP (130–139/85–89 mmHg) was not associated with the risk of all-cause and CHD mortality. Notably, a meta-analysis conducted by Brunström et al. [45] showed that regarding primary prevention, management of hypertension (baseline SBP ≥ 140 mmHg) was associated with reduced risk of death and major CVD outcomes; however, it lacked effect if baseline SBP was below 140 mmHg.

## Strengths and limitations

The current study has a number of strengths. First, we evaluated the association of four MetS definitions with the risk of SCD by a prospective population-based cohort of Iranian men and women aged ≥ 40 years over two decades of follow-up. Second, we used standardized protocols to measure risk factors. On the other hand, our study has several limitations. As we were unable to validate some of the deaths coded as SCD, there is the chance for inclusion of false-positive SCD events that might have been deaths due to other causes (e.g., cerebral hemorrhage or pulmonary embolisms). Second, although there were 182 SCD cases during the 17.9 years of follow-up, we did not have adequate statistical power to assess some clinical subgroups (e.g., age and sex). In the TLGS, those who survived sudden cardiac arrest were not included as cases since the definition was based on the mortality events; moreover, unfortunately, the rate of successful CPR with discharge from hospital in Iran is too low (ranged from 5 to 12%) [46–49] to have a considerable effect on the number of events in our data set. Finally, the current study was conducted on a sample of the residents of Tehran; thus, our findings might not be extrapolated to the whole country, especially the rural area.

## Conclusions and relevance

Results from this study indicate that the constellation of MetS (based on the JIS, IDF, and WHO, but not ATP III criteria) is a marker for identifying individuals at higher risk for SCD; however, not independent of its components. We also showed that among MetS components, abdominal obesity (using the population-specific cutoff point of WC), high glucose component according to the JIS/IDF MetS definitions, and BP ≥ 140/90 mmHg (WHO definition) were significantly associated with the risk of SCD. Our results suggest that lifestyle interventions focusing on the three above main MetS components through promoting healthy, low-calorie, low-salt diets and encouraging physical activity to correct abdominal obesity and dysglycemia status might potentially reduce the catastrophic events of SCD.

## Supplementary Information

**Additional file 1: Figure S1.** The flowchart of the participant selection process in the current study.

**Additional file 2: Figure S2.** Distribution of the causes of death in the total population.

**Additional file 3: Table S1.** Baseline characteristics of the responders and non-responders.

**Additional file 4: Table S2.** Baseline characteristics of the study population stratified by the incidence of sudden cardiac death (population for the WHO criteria): Tehran Lipid and Glucose Study (1999-2018)

## Data Availability

The datasets used and analyzed during the current study are available from the corresponding author upon reasonable request.

## Abbreviations

MetS: Metabolic syndrome
SCD: Sudden cardiac death
CVD: Cardiovascular disease
T2D: Type 2 diabetes
CHD: Coronary heart disease
CPR: Cardiopulmonary resuscitation
MENA: Middle East and North Africa
BP: Blood pressure
FPG: Fasting plasma glucose
TLGS: Tehran Lipid and Glucose Study
NCD: Noncommunicable disease
WHO: World Health Organization
FH-CVD: Family history of premature CVD
WC: Waist circumference
HC: Hip circumference
BMI: Body mass index
WHR: Waist to hip ratio
2 h-PG: 2-Hour post-challenge plasma glucose
RHR: Resting heart rate
TG: Triglycerides
HDL-C: High-density lipoprotein cholesterol
IHD: Ischemic heart disease
MI: Myocardial infarction
CVA: Cerebrovascular accident
IDF: International Diabetes Federation
ATP III: Adult Treatment Panel III
JIS: Joint Interim Statement
EGIR: European Group for the Study of Insulin Resistance
SBP: Systolic blood pressure
DBP: Diastolic blood pressure
K-NHIS: Korean National Health Insurance Service
ARIC: Atherosclerosis Risk in Communities
KIHD: Kuopio Ischemic Heart Disease
SD: Standard deviation
HR: Hazard ratio
RR: Relative risk
CI: Confidence interval
