# Supplementary material for: The clinical value of metabolic syndrome and its components with respect to sudden cardiac death using different definitions: Two decades of follow-up from the Tehran Lipid and Glucose Study
Source: Cardiovasc Diabetol. 2022 Dec 3;21:269. doi: 10.1186/s12933-022-01707-1 (PMC9719125; doi:10.1186/s12933-022-01707-1)
Supplement: Supplementary file 1 — Additional file 1: Figure S1. The flowchart of the participant selection process in the current study. [file 12933_2022_1707_MOESM1_ESM.docx]

6,295 participants aged ≥40 years were recruited. [5,282 recruited from **phase 1** (1999-2002) and 1,013 recruited from **phase 2** (2002-2005)]

5079 participants^† ††^ remained eligible for the analysis.

569 individuals with prevalent CVD at baseline were excluded.

Leading to 5,726 participants aged ≥40 years.

196 individuals were excluded due to missing data on MetS components or covariates.

451 individuals without any follow-up data were excluded.

Non-responders

**Figure S1. The flowchart of the participant selection process in the current study.**

^†^ For WHO criteria of the MetS, further 139 individuals were excluded due to missing data on 2h-PG, leading to 4940 eligible participants.

^††^ This number included those with SCD (n=182), non-SCD death (n=597), loss to follow-up or those who left the district (n=675), and those alive at the end of study, March 2018 (n=3,625)
