# Supplementary figures and images for: The clinical value of metabolic syndrome and its components with respect to sudden cardiac death using different definitions: Two decades of follow-up from the Tehran Lipid and Glucose Study
Source: Cardiovasc Diabetol. 2022 Dec 3;21:269. doi: 10.1186/s12933-022-01707-1 (PMC9719125; doi:10.1186/s12933-022-01707-1)

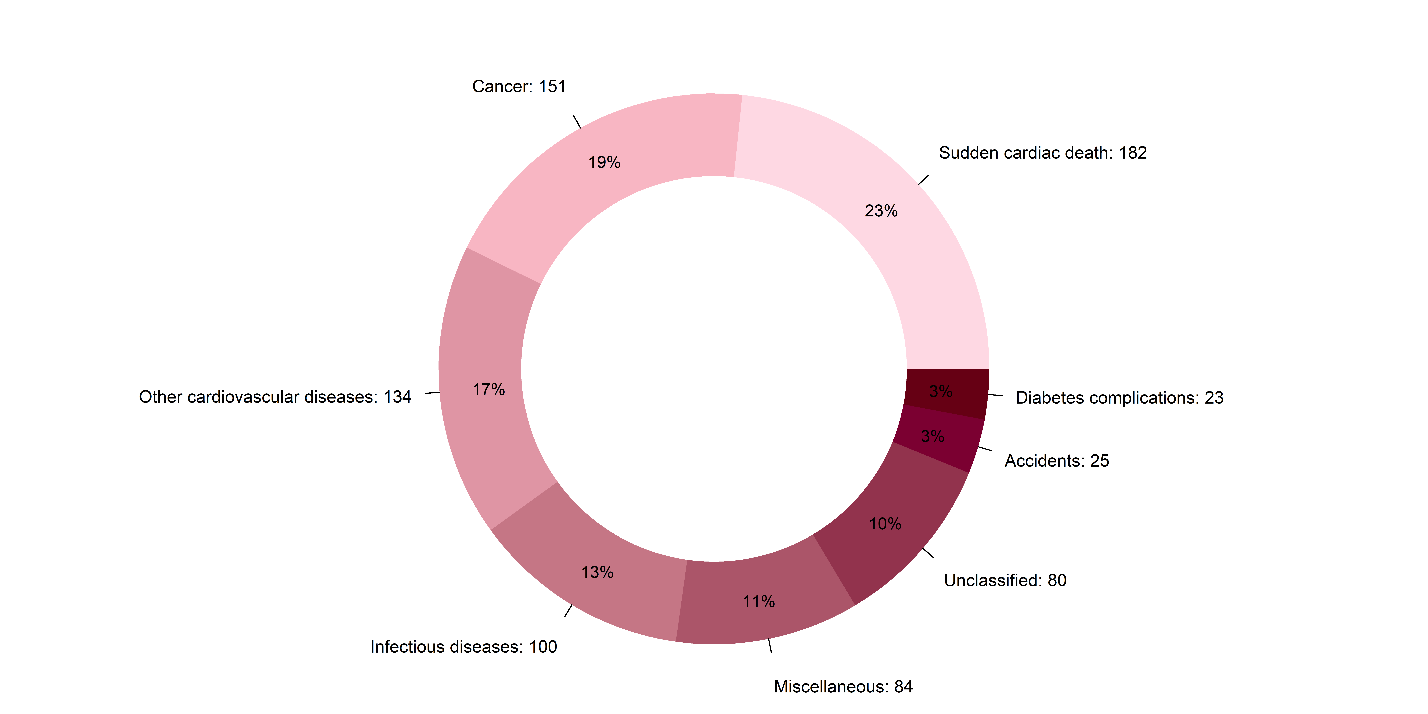


- **Figure S2 Distribution of the causes of death in the total population**

Supplement: Supplementary file 2 — Additional file 2: Figure S2. Distribution of the causes of death in the total population. [file 12933_2022_1707_MOESM2_ESM.docx]
