# Supplementary material for: The clinical value of metabolic syndrome and its components with respect to sudden cardiac death using different definitions: Two decades of follow-up from the Tehran Lipid and Glucose Study
Source: Cardiovasc Diabetol. 2022 Dec 3;21:269. doi: 10.1186/s12933-022-01707-1 (PMC9719125; doi:10.1186/s12933-022-01707-1)
Supplement: Supplementary file 3 — Additional file 3: Table S1. Baseline characteristics of the responders and non-responders. [file 12933_2022_1707_MOESM3_ESM.docx]

| Table S1. Baseline characteristics of the responders and non-responders. | | | |
| --- | --- | --- | --- |
|  | **Responders** | **Non-responders†** | **P-value** |
| Number of participants | 5079 | 647 |  |
| Continuous variables, Mean ± SD | | | |
| Age (year) | 53.63 ± 9.94 | 55.00 ± 11.00 | <0.01 |
| BMI (kg/m^2^) | 27.88 ± 4.59 | 27.96 ± 4.64 | 0.69 |
| WC (cm) | 92.71 ± 11.22 | 92.42 ± 10.80 | 0.56 |
| WHR | 0.91 ± 0.08 | 0.91 ± 0.07 | 0.58 |
| SBP (mmHg) | 126.11 ±20.90 | 127.40 ± 21.79 | 0.14 |
| DBP (mmHg) | 80.10 ±11.43 | 80.50 ± 11.04 | 0.40 |
| RHR (beat/min) | 78.34 ± 11.44 | 77.75 ± 11.98 | 0.21 |
| FPG (mg/dl) | 105.18 ± 39.38 | 106.63 ± 40.38 | 0.42 |
| 2h-PG (mg/dl) | 131.23 ± 64.51 | 133.80 ± 61.46 | 0.42 |
| HDL-C (mg/dl) | 41.67 ± 10.95 | 42.67 ± 10.80 | 0.05 |
| TG (mg/dl) | 165 (115-233)* | 155.5 (115-223.5)* | 0.25 |
|  | | | |
| Categorical variables, number (%) | | | |
| Men | 2,294 (45.17) | 263 (40.65) | 0.03 |
| Current smoking, yes | 803 (15.81) | 125 (19.32) | 0.07 |
| Family History of premature CVD, yes | 932 (18.35) | 119 (18.39) | 0.98 |
| Glucose-lowering drug use, yes | 335 (6.60) | 62 (9.58) | <0.01 |
| Anti-hypertensive drug use, yes | 569 (11.20) | 86 (13.29) | 0.11 |
| Lipid-lowering drug use, yes | 252 (4.96) | 51 (7.88) | <0.01 |
| Abbreviations: SD, standard deviation; BMI, body mass index; WC, waist circumference; WHR, waist to hip ratio; SBP, systolic blood pressure; DBP, diastolic blood pressure; RHR, resting heart rate; FPG, fasting plasma glucose; 2h-PG, 2-hour post-challenge glucose; HDL-C, high-density lipoprotein cholesterol; TG, triglycerides; CVD, cardiovascular disease.  *Data presented as median (IQR).  †Non-responders included those with missing data on MetS components/covariates or those with no follow-up data. | | | |
