# Supplementary material for: The clinical value of metabolic syndrome and its components with respect to sudden cardiac death using different definitions: Two decades of follow-up from the Tehran Lipid and Glucose Study
Source: Cardiovasc Diabetol. 2022 Dec 3;21:269. doi: 10.1186/s12933-022-01707-1 (PMC9719125; doi:10.1186/s12933-022-01707-1)
Supplement: Supplementary file 4 — Additional file 4: Table S2. Baseline characteristics of the study population stratified by the incidence of sudden cardiac death (population for the WHO criteria): Tehran Lipid and Glucose Study (1999-2018) [file 12933_2022_1707_MOESM4_ESM.docx]

| Table S2. Baseline characteristics of the study population stratified by the incidence of sudden cardiac death (population for the WHO criteria): Tehran Lipid and Glucose Study (1999-2018) | | | |
| --- | --- | --- | --- |
|  | **Without SCD** | **With SCD** | **P-value** |
| Number of participants | 4769 | 171 |  |
| Continuous variables, Mean ± SD |  | | |
| Age (year) | 53.18 ± 9.66 | 62.01 ± 9.75 | <0.01 |
| BMI (kg/m^2^) | 27.92 ± 4.56 | 27.69 ± 5.02 | 0.52 |
| WC (cm) | 92.69 ± 11.16 | 95.28 ± 11.83 | <0.01 |
| WHR | 0.91 ± 0.08 | 0.96 ± 0.08 | <0.01 |
| SBP (mmHg) | 125.67 ± 20.59 | 138.55 ± 25.83 | <0.01 |
| DBP (mmHg) | 80.04 ± 11.29 | 83.26 ± 14.28 | <0.01 |
| RHR (beat/min) | 78.38 ± 11.38 | 78.37 ± 12.87 | 0.99 |
| FPG (mg/dl) | 104.62 ± 38.34 | 129.56 ± 65.46 | <0.01 |
| 2h-PG (mg/dl) | 130.50 ± 62.64 | 155.97 ± 107.83 | <0.01 |
| HDL-C (mg/dl) | 41.69 ± 10.92 | 41.40 ± 11.27 | 0.73 |
| TG (mg/dl) | 165 (116-234)* | 170 (121-235)* | 0.62 |
|  |  | | |
| Categorical variables, number (%) |  | | |
| Men | 2119 (44.43) | 113 (66.08) | <0.01 |
| Current smoking, yes | 737 (15.45) | 38 (22.22) | 0.02 |
| Family History of premature CVD, yes | 888 (18.62) | 26 (15.20) | 0.26 |
| Glucose-lowering drug use, yes | 302 (6.33) | 33 (19.30) | <0.01 |
| Anti-hypertensive drug use, yes | 506 (10.63) | 41 (23.90) | <0.01 |
| Lipid-lowering drug use, yes | 234 (4.91) | 12 (7.02) | 0.21 |
| Abbreviations: SCD, sudden cardiac death; SD, standard deviation; BMI, body mass index; WC, waist circumference; WHR, waist to hip ratio; SBP, systolic blood pressure; DBP, diastolic blood pressure; RHR, resting heart rate; FPG, fasting plasma glucose; 2h-PG, 2-hour post-challenge glucose; HDL-C, high-density lipoprotein cholesterol; TG, triglycerides; CVD, cardiovascular disease; WHO, World Health Organization.  *Data presented as median (IQR). | | | |
